# Intraoperative Ultrasound in Chiari 1 Decompression: Clarity or Confusion? A Systematic Review

**DOI:** 10.1227/neu.0000000000003818

**Published:** 2025-10-23

**Authors:** Thomas Petutschnigg, Andreas Raabe, Philippe Schucht, Katharina Lutz

**Affiliations:** Department of Neurosurgery, Inselspital, Bern University Hospital, University of Bern, Bern, Switzerland

**Keywords:** Chiari 1 malformation, Posterior fossa decompression, Foramen magnum decompression, Intraoperative ultrasound, Doppler

## Abstract

**BACKGROUND AND OBJECTIVES:**

Patients with symptomatic Chiari malformation type 1 (CM-1) usually undergo either bone-only posterior fossa decompression (PFD) or a more invasive procedure involving dural expansion with or without tonsillar resection (PFD+). PFD+ may be more effective but it carries a higher risk of complications. Intraoperative ultrasound (iUS) may help determine whether PFD alone is sufficient, although specific criteria and its role in surgical decision making are not yet defined.

**METHODS:**

A systematic review was conducted following Preferred Reporting Items for Systematic Reviews and Meta-Analysis guidelines, using search terms related to iUS, intraoperative sonography, and CM-1. Extracted data included demographics, surgical methods, iUS criteria, and outcomes. Conversion rates and the positive predictive values (PPVs) were calculated to quantify iUS effectiveness. Conversion rate was defined as the proportion of cases requiring secondary PFD+ after initial PFD. PPV was based on the number of successful PFDs relative to all initial PFDs. Risk of bias was evaluated using the Newcastle-Ottawa scale.

**RESULTS:**

From 202 initial articles, 1 prospective and 8 retrospective studies were included, covering 844 pediatric and adult patients. According to the Newcastle-Ottawa scale, 1 study had a low, 3 moderate, and 5 high risk of bias. To assess decompression sufficiency, 8 studies used qualitative iUS criteria, whereas 1 used quantitative thresholds. Follow-up time ranged from 1 to 48 months, with varied outcome measures. Conversion rates ranged from 0% to 16% and PPV from 0.857 to 1.000, depending on the study. Conversion surgery rates were comparable with reoperation rates reported from studies without iUS.

**CONCLUSION:**

The available evidence does not yet support iUS as a robust testing method to inform intraoperative decision making regarding the extent of decompression, due to the heterogeneity of its application, and the absence of standardized assessment criteria. We propose a reporting framework incorporating all currently reported criteria to induce standardization and guide further research.

**ABBREVIATIONS:**
CM-1: Chiari malformation type 1
FP: false positive
FU: follow-up
iUS: intraoperative ultrasound
PFD: posterior fossa decompression
TP: true positive.

Chiari malformation type 1 (CM-1) is defined as overcrowding at the craniocervical junction with tonsillar herniation.^1-4^ Its etiology remains incompletely understood. Genetic predisposition, developmental abnormalities, craniocervical instability, and disturbances in cerebrospinal fluid (CSF) dynamics have been identified as potential contributing factors.^5-10^ Although CM-1 prevalence is estimated at 0.1%, incidental findings meeting radiological criteria are becoming more common because of the increased use of imaging.^11^

Surgical intervention is necessary for symptomatic CM-1 patients,^2,12,13^ but there is ongoing debate regarding the optimal technique and the extent of decompression. Bone-only posterior fossa decompression (PFD) and dural expansion with or without tonsillar resection (PFD+) each present distinct advantages and risks.^2,14-22^ However, PFD is associated with a 1.24-fold higher risk of inadequate decompression, PFD+ is more invasive and has a 4.5-fold higher complication rate.^16^

Although conventional imaging, such as computed tomography or magnetic resonance imaging, remains essential for diagnosis, surgical planning, and follow-up (FU), it lacks dynamic assessment capabilities.^23^ Intraoperative ultrasound (iUS) has emerged as a valuable tool,^24-38^ offering cost-effective intraoperative imaging. Technological advancements have improved ultrasound image quality, enhancing its utility in neurosurgical applications.^39^

This systematic review critically examines the existing literature on iUS in CM-1 surgery, with a focus on evaluating its efficacy in guiding tailored surgical strategies. We assess the potential of iUS to differentiate between the need for PFD or more invasive techniques, while also addressing the limitations and uncertainties in its application. The aim of this review was to provide a comprehensive evaluation of iUS as a decision-making tool and its overall impact on surgical outcomes, highlighting areas where further research is needed.

## METHODS

### Literature Search Strategy

The review was performed in accordance with the Preferred Reporting Items for Systematic Reviews and Meta-Analysis guidelines.^40^ Initially conceived as an exploratory study, it evolved into a structured review based on emerging findings. As a result, the study was not prospectively registered in a systematic review registry. To ensure a comprehensive assessment, we conducted a structured search on July 7, 2024, using PubMed/MEDLINE and Ovid/Embase with the following combined terms: (Chiari malformation) AND ([intraoperative ultrasound] OR [intraoperative sonography]). We reviewed all identified abstracts, studies, and citations. In addition, we manually searched the reference lists of the retrieved articles for further relevant publications.

### Ethics

No ethical approval was necessary for this systematic review, as it did not involve human or animal subjects and was limited to the evaluation of previously published studies.

### Inclusion and Exclusion Criteria

All eligible records were systematically assessed using predefined inclusion and exclusion criteria. We included studies reporting (1) all age groups with a confirmed diagnosis of CM-1, where (2) iUS was used in determining whether to open the dura mater, (3) studies reporting at least a brief description of the iUS criteria that were considered in the decision-making process. Studies had to report (4) clinical outcomes, (5) reoperation rates, and the (6) FU time.

We excluded animal studies, editorials, and opinion letters, as well as studies on any other types of Chiari malformations. When institutions had published duplicate studies with accumulating numbers of patients or increased durations of FU, only the most complete or most recent reports were included.

### Study Selection, Data Collection, and Extraction

Studies that met the predefined inclusion criteria were then selected for full-text review, and each study was assessed independently by 2 authors (T.P. and K.L.) to determine eligibility. Standardized tables were used to extract the following information from the abstract or full text: author(s) and year, study type, demographics, patient number, surgical methods, iUS measures, FU duration, outcome, complications, and reoperations. The surgical techniques were dichotomized into bone-only PFD and extended PFD, which included any form of dura opening, duraplasty, and/or tonsillar PFD+.

### Conversion Rate and PPV

To objectify the benefit of iUS, we introduced the concept of “conversion surgery,” defined as revision surgery from PFD to PFD+ at any point during FU because of symptom persistence or worsening. To determine conversion surgeries, we reviewed all reported reoperations and identified those classified as conversion surgeries, excluding procedures performed because of complications such as wound infections or CSF leaks.

For our analysis, we defined a positive iUS result as one suggesting that PFD would be sufficient. Conversely, iUS findings indicating the need for PFD+ were considered negative test results. To quantify the diagnostic yield of iUS under this framework, we constructed a 2-by-2 contingency table (Table 1). In this context, we defined true positives (TPs) as cases where iUS guidance led to PFD and no further conversion surgery was needed during FU. False positives (FPs) were cases in which iUS suggested PFD was sufficient, but the patient later required conversion to PFD+.

**TABLE 1.** 2-By-2 Contingency Table

	PFD sufficient	PFD+ sufficient
iUS: PFD (test positive)	TP	FP
iUS: PFD+ (test negative)	FN	TN

FN, false negative; FP, false positive; iUS, intraoperative ultrasound; PFD, bone-only posterior fossa decompression; PFD+, posterior fossa decompression with duraplasty with or without tonsillar shrinkage, TN, true negative; TP, true positive.

iUS test positive means PFD seemed adequate. iUS test negative, means PFD+ seemed necessary. TP: iUS suggested PFD, no conversion needed; FP: iUS suggested PFD, patient required secondary conversion to PFD+; TN: iUS suggested PFD+, no further decompression (already maximum decompression); FN: iUS suggested PFD+, but PFD might have sufficed (ie, potential overdecompression, impossible to assess).

This allowed for the calculation of the conversion rate (or false discovery rate):

$$\text{Conversion}\;\text{rate}=\frac{\text{FP}}{\text{TP}+\text{FP}}$$

In addition, we assessed the diagnostic accuracy of iUS by calculating the positive predictive values (PPVs), which quantifies the proportion of iUS-guided PFD cases that were successful.

$$\text{PPV}=\frac{\text{TP}}{\text{TP}+\text{FP}}$$

It is impossible to determine how many patients underwent PFD+ even though PFD might have sufficed. Therefore, false negatives and true negatives are inherently unquantifiable, making sensitivity and specificity analysis not feasible.

Statistical analyses were performed using SPSS software (IBM, version 28), whereas graphs were generated using Excel (Microsoft, version 16.94). To demonstrate the reliability of our findings, we reported 95% CIs, calculated using the Wilson score method.

### Risk of Bias

The risk of bias in the studies was evaluated using the Newcastle-Ottawa scale.^41^ The scale ranges from 0 to 9 points: 7 to 9 points indicate a low risk of bias, 4 to 6 points a moderate risk, and 0 to 3 points a high risk of bias.

## RESULTS

### Included Studies

**FIGURE 1.**
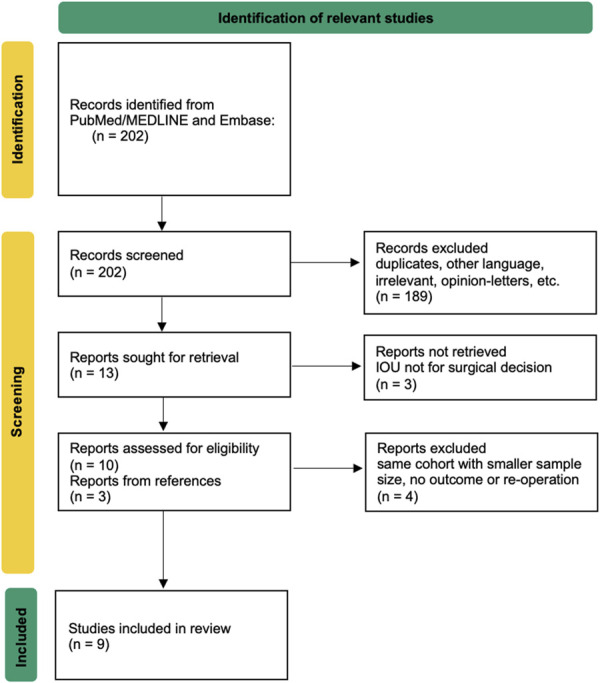
Flowchart showing the selection process for study inclusion.

The study selection process is depicted in Figure 1. We conducted a comprehensive review of 202 studies identified through an electronic search. After applying our inclusion and exclusion criteria, 9 studies were selected for analysis (Table 2). Our search did not yield any randomized controlled trials or studies that exclusively compared patient groups with and without adjunct iUS. Most of the studies (8) were retrospective analyses,^26-30,32-34^ with only 1 prospective study.^31^ The patient populations in the included studies varied. Five studies reported only on pediatric patients,^26-28,30,34^ 1 included both children and adults,^29^ and 3 studies reported solely on adult patients.^31-33^

**TABLE 2.** Overview of Patient and Study Characteristics of the Included Studies

Authors and year	Study type	Cohort type	No. of patients	No. of patients with syrinx (%)	Age (y)	PFD (%)	PFD+ (%)	No. of patients who underwent iUS (%)	iUS category	iUS criteria for sufficient decompression	Clinical outcome	Mean FU (mo)	Reoperation(s) (%)	Conversion(s)	Conversion rate (%)
Navarro et al,^26^ 2004	RS	Pediatric	96	41 (43)	0.5-18	56 (51)	53 (49)	61 (64)	Qualitative	Syrinx → duraplasty	Improved vs unimproved	28	8 (14) PFD	6	11
										No syrinx → iUS: sufficient subarachnoid space and tonsillar pulsations			5 (9) PFD+		
Yeh et al,^27^ 2006	RS	Pediatric	130	26 (20)	0.75-18	44 (34)	86 (66)	130 (100)	Qualitative	CSF space anterior to the brainstem and dorsal to the cerebellar tonsils and no evidence of piston-like effect	Notable improvement of symptoms	20	5 (11) PFD	5	11
													2 (2) PFD+		
McGirt et al,^28^ 2008	RS	Pediatric	256	69 (27)	10	116 (45)	140 (55)	256 (100)	Qualitative	Subarachnoid space ventral and dorsal to the cerebellum and tonsils, no evidence of piston-like effect	Symptom persistence vs recurrence	29	9 (8) PFD	7	6
													10 (11) PFD+		
Cui et al,^29^ 2011	RS	Mixed	20	18 (90)	45	1 (5)	19 (95)	20 (100)	Qualitative	Bidirectional flow of the CSF	Improvement, stabilization, deterioration	12	0 (0) PFD	0	0
													0 (0) PFD+		
Narenthiran et al,^30^ 2015	RS	Pediatric	19	14 (74)	11	8 (42)	11 (58)	19 (100)	Qualitative	Tonsillar and/or CSF pulsation	Better, same and worse	12	1 (13) PFD	1	13
													2 (18) PFD+		
Brock et al,^31^ 2017	PS	Adult	49	17 (47)	48	36 (73)	13 (27)	49 (100)	Quantitative	CSF flow velocity ≥ 3 cm/s	NRS, better, same, worse and SF-36	12	6 (16) PFD	6	16
													3 (20) PFD+		
Dherijha et al,^32^ 2021	RS	Adult	57	21 (31)	47	54 (95)	3 (5)	57 (100)	Qualitative	Pulsatility of the tonsils and space ventral/dorsal cisterns	CCOS	38	7 (13) PFD	7	13
													1 (33) PFD+		
Jha et al,^33^ 2024	RS	Adult	15	9 (60)	31 PFD	7 (47)	8 (53)	15 (100)	Qualitative	CSF pulsatile flow and tonsillar movement	CCOS, CSS	1^a^	0 (0) PFD	0	0
					52 PFD+								0 (0) PFD+		
Venanzi et al,^34^ 2024	RS	Pediatric	202	60 (28)	8	119 (60)	83 (40)	151 (72)	Qualitative	Syrinx → duraplasty	CCOS	48	3 (3) PFD	3	3
										No syrinx → iUS: CSF layer behind the tonsils and anterior-posterior pattern of tonsillar pulsation			3 (4) PFD+		

CCOS, Chicago Chiari Outcome Scale; CSF, cerebrospinal fluid; CSS, clinical sign score; FU, follow-up; iUS, intraoperative ultrasound; NRS, numeric rating scale; PFD, bone-only posterior fossa decompression; PFD+, posterior fossa decompression with duraplasty with or without tonsillar shrinkage; PS, prospective; RS, retrospective; SF-36, short form-36.

a Minimum duration with unclear full length of FU duration.

 Only 1 study was classified as having a “low” risk of bias,^31^ 3 were rated as “moderate,”^26-28^ and 5 were identified as having a “high” risk of bias.^29,30,32-34^ The majority did not provide detailed information on patient selection processes, and only a few adjusted for confounding factors. Clinical and radiographic outcomes were assessed in all studies, but details on duration and completeness of FU were generally lacking or not provided consistently. The results are summarized in Table 3.

**TABLE 3.** Methodological Quality Assessment Based on the NOS

Authors and year	Selection	Comparability	Outcome	Total
Navarro et al,^26^ 2004	2	1	2	5
Yeh et al,^27^ 2006	2	1	2	5
McGirt et al,^28^ 2008	2	2	2	6
Cui et al,^29^ 2011	1	1	1	3
Narenthiran et al,^30^ 2015	1	1	1	3
Brock et al,^31^ 2017	2	2	3	7
Dherijha et al,^32^ 2021	1	0	2	3
Jha et al,^33^ 2024	1	1	1	3
Venanzi et al,^34^ 2024	1	1	2	3

NOS, Newcastle-Ottawa scale.

9-7 points: low risk of bias, 6-4 points: moderate risk of bias, 3-0 points: high risk of bias.

### Surgeries and iUS Utilization

Nine studies involving 844 patients (range: 15-256 patients/study) were analyzed (Table 2). iUS was used for all patients in 7 studies, whereas, in 2 studies, it was only applied for patients without syringomyelia.^26,34^ Thus, a total of 758 patients were evaluated with iUS. Overall, 275 patients (33%) presented preoperatively with some form of syringo(hydro-)myelia. In total, 441 PFD and 416 PFD+ surgeries were performed, all in prone position with rigid head fixation.

iUS was consistently used after suboccipital craniectomy/foramen magnum decompression, before dura opening. None of the studies used iUS after dura opening. The evaluation of iUS findings varied: 2 studies involved neuroradiologists or neurosonographers,^28,31^ whereas 3 relied on the responsible neurosurgeons.^28,33,34^ The other 4 studies did not specify the evaluator.^26,29,30,32^ Duraplasty techniques, when applied, showed variations in dural incision, graft type, and intradural manipulation.

### Applied iUS Criteria

The reported iUS criteria can be categorized as follows: studies using qualitative criteria (8) vs studies using quantitative measurements (1). Within the qualitative group, 7 studies considered anatomic spatial conditions as the primary criterion,^26-30,32,34^ whereas 1 study focused on the presence of a physiological bidirectional CSF flow.^29^ The sole study with quantitative measurements assessed CSF flow velocity thresholds.^31^

#### Qualitative Criteria

The 7 studies that primarily assessed the spatial conditions applied similar but not identical criteria. Navarro et al^26^ and Venanzi et al^34^ evaluated only patients without a syrinx to choose between PFD and PFD+. If physiological pulsation of the tonsils and adequate subarachnoid space ventral and dorsal the brainstem was found, the dura was left intact. This led to a PFD rate of 54%^26^ and 60%,^34^ respectively. The largest included cohort, with 256 patients, was studied by McGirt et al^28^ who assessed for sufficient space in front of and behind the cerebellum, as well as the absence of piston-like pulsations. They found that PFD alone doubled symptom recurrence risk as compared with PFD+ in patients with tonsillar herniation beyond C1 (odds ratio 2.05, 95% CI 1.04-3.78, *P* = .034), whereas with herniation above C1, iUS confirmed adequate decompression reliably (odds ratio 1.16, 95% CI 0.33-4.05, *P* = .41). Yeh et al^27^ left the dura closed if the space anterior to the brainstem, dorsal to the cerebellar tonsils, was sufficient and no evidence of a piston-like effect was present. They reported that PFD was performed in 34% of cases (as-treated). Narenthiran et al^30^ opened the dura in the absence of CSF or tonsillar pulsation in the iUS. Dherijha et al^32^ left the dura intact when sufficient space was present in the ventral and dorsal cisterns and the tonsils pulsated freely. With 15 patients, Jha et al's^33^ was the smallest cohort included in this review. Their iUS criteria were vaguely defined, referring to adequate CSF flow and tonsillar movement. Cui et al^29^ measured the CSF flow velocity infracerebellar, yet they based their decision to leave the dura closed solely on the presence of a “physiological cardiac and ventilation-dependent bidirectional CSF flow.”

#### Quantitative Criteria

Brock et al,^31^ whose study was the only one that applied a quantitative criterion, prospectively assessed a CSF flow velocity threshold as the decisive criterion. After performing bone decompression and dural delamination, CSF flow was assessed and the infracerebellar space was measured in 3 dimensions for each patient. If the velocity of the CSF was 3 cm/s or more in the retrocerebellar space, the procedure was concluded without opening the dura. They found no significant difference in the infracerebellar space between PFD and PFD+ group (Table 2).

#### FU and Outcome Measures

Reported FU durations varied widely across the included studies. The shortest reported FU was a minimum of 1 month,^33^ although the authors stated a longer FU period was achieved, they did not further specify the exact duration. By contrast, the longest reported mean FU was 48 months.^34^ A wide variety of outcome measures were reported, as listed in Table 2.

### Reoperation and Conversion Surgeries

**FIGURE 2.**
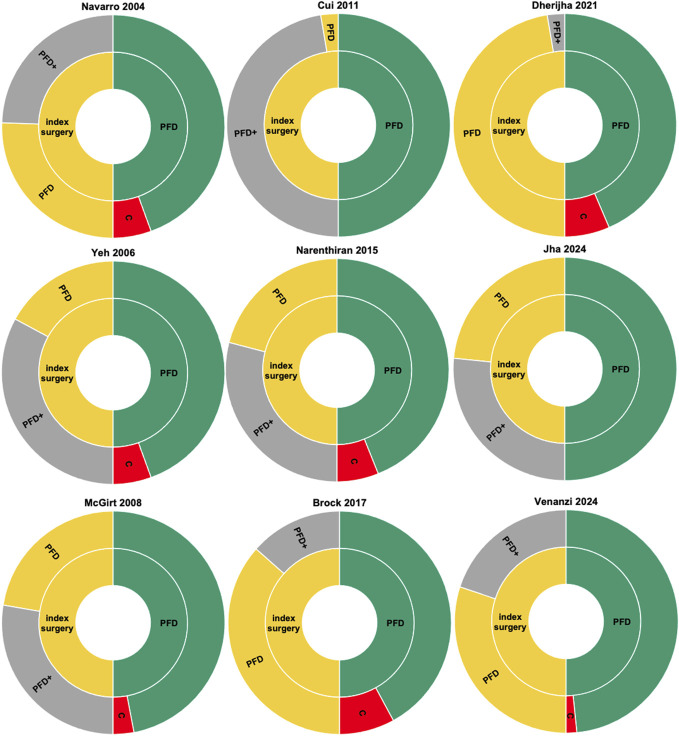
Sunburst graph for each study. Left half shows the PFD (yellow) and PFD+ (gray) rates of index surgeries. Right half illustrates the proportion of successful PFD (green) and the conversion rate (red/C) during FU. FU, follow-up; PFD, bone-only posterior fossa decompression; PFD+, posterior fossa decompression with duraplasty with or without tonsillar shrinkage.

The cumulative reoperation rate ranged from 0% to 16% for PFD and from 0% to 33% for PFD+. The published data from all studies enabled the calculation of conversion surgeries and rates. In total, 35 of 844 patients (5%) had to convert from PFD to PFD+ at some point, because of either lack of clinical improvement or clinical deterioration, but the clinical end points were not uniformly defined. Within all studies, the conversion rate ranged from 0% to 16% (Table 2, Figure 2).

### PPV of iUS Criteria

The analysis of the included studies revealed considerable variability in the diagnostic value of iUS. The highest PPV was calculated for the studies by Cui et al^29^ and Jha et al.^33^ Based on their results, we estimated the PPV at 1.000, with 95% CIs of 0.207 to 1.000 and 0.676 to 1.000, respectively. By contrast, we found that the study by Brock et al^31^ had the lowest PPV, at 0.8337 (95% CI 0.7681-0.921). A summary of all calculated PPV values and CI is provided in Table 4.

**TABLE 4.** Estimated Diagnostic Value of iUS Criteria for Each Study Using the PPV

Authors and year	TP	FP	PPV	95% CI
Navarro et al,^26^ 2004	50	6	0.893	0.785-0.950
Yeh et al,^27^ 2006	39	5	0.886	0.760-0.950
McGirt et al,^28^ 2008	109	7	0.940	0.881-0.970
Cui et al,^29^ 2011	1	0	1.000	0.207-1.000
Narenthiran et al,^30^ 2015	7	1	0.875	0.529-0.978
Brock et al,^31^ 2017	30	6	0.833	0.681-0.921
Dherijha et al,^32^ 2021	47	7	0.870	0.756-0.936
Jha et al,^33^ 2024	7	0	1.000	0.646-1.000
Venanzi et al,^34^ 2024	116	3	0.975	0.928-0.991

FP, false positive; FU, follow-up; iUS, intraoperative ultrasound; PFD, bone-only posterior fossa decompression; PFD+, posterior fossa decompression with duraplasty with or without tonsillar shrinkage; PPV, positive predictive value; TP, true positive.

TP: PFD without the need for conversion, FP: PFD with the conversion to PFD+ during FU.

## DISCUSSION

Determining whether PFD alone is sufficient for CM-1 is crucial to avoiding unnecessary risk from PFD+ while preventing the need for conversion surgery. This underscores the need for a reliable tool to balance these risks. We review the current use of iUS in CM-1 and critically assess its application. The literature lacks consistent criteria and robust comparative studies, limiting definitive conclusions. However, iUS clarifies or confuses remains unanswered.

### Assessing Whether There is “Enough Space”

Most of the included studies relied on qualitative criteria to assess the space at the craniocervical junction, but these criteria are often described in different ways, leading to redundancy. Commonly used determinants were “sufficient subarachnoid space,”^26^ “CSF layer behind the tonsils,”^34^ “pulsatility of the tonsils,”^30^ “absence of piston-like pulsation,”^27,28^ or “bidirectional CSF flow,”^29^ but none of the studies provided specific quantitative measurements, for example, metrics of subarachnoid space distances at the craniocervical junction. Many studies referred to the work of Oldfield et al^24^ to explain the phenomenon of piston-like pulsations, although this remains a subjective interpretation. Generally, the overlapping criteria, articulated in varying ways, emphasize the need for standardization in future trials, including quantitative, age-specific reference values. Such standardization could reduce redundancy and inter-rater variability, improve the consistency of findings, and provide guidelines for surgical decision making.

### The CSF Flow Velocity

The only study in the literature that relied on CSF flow velocities was conducted by Brock et al.^31^ They applied a CSF flow velocity threshold of ≥3 cm/s, as postulated by Milhorat and Bolognese,^8^ to determine whether the dura could remain closed. However, this criterion demonstrated the lowest PPV among the included studies, as 1 in 6 patients required conversion surgery at some later point. In contrast to blood, CSF has a low content of cells, which ultimately results in a reduced echo signal. In addition, CSF circulates at quite low velocities and flows through rather irregular spaces compared, for example, with blood in vessels, resulting in nonhomogeneous and nonlaminar flow.^8,28^ These difficulties can be partially overcome by adjusting the frequency to increase spatial resolution, appropriately increasing the gain to amplify the signal, and angling the probe relative to the flow direction.^29,39^ Fundamental physical principles, a cranial-caudal CSF flow and an overcrowded foramen magnum, suggest the CSF flow velocity below the blockage to be lower than in healthy individuals. Yet, the location chosen to measure the CSF flow is not consistent in the literature. While referencing Milhorat and Bolognese,^8^ Brock et al^31^ measured retrocerebellar at the point of maximal obstruction, whereas Milhorat and Bolognese^8^ and Cui et al^29^ specified that they measured infracerebellar dorsal to the spinal cord. This underscores the heterogeneity of iUS application.

### Does iUS Provide Clarity or Confusion?

Although all studies concluded that iUS was beneficial, they failed to provide a clear definition of success or any statistical validation.^26-34^ Moreover, McGirt et al^28^ found that iUS was unreliable in a subgroup of patients with tonsillar herniation beyond the C1 lamina. Across all studies, PFD was deemed iUS-sufficient in about half of the patients. However, the PFD rate varied significantly (Figure 2), probably due to the heterogeneous cohorts and to the diverse iUS criteria. Given the available data, assessing diagnostic value through PPV calculations was a feasible approach. Nevertheless, evaluating the efficacy of different iUS criteria is not trivial for Chiari surgery, as PFD+ is an extension of PFD. This sequential relationship complicates analysis, making it difficult to isolate the effects of the individual interventions and impossible to determine how many patients underwent PFD+ even though PFD might have already sufficed. Hence, the sensitivity and specificity cannot be calculated, limiting the diagnostic and decision-making accuracy of iUS. Only a gold standard tool capable of determining the true necessity of duraplasty in each case could resolve this issue, yet such a tool does not exist.

Even though bidirectional flow^29^ demonstrated a high PPV, these results should be interpreted with caution because of the small sample size and the wide CIs. Studies that evaluated tonsillar movement and the subarachnoid space around the foramen magnum reported conversion rates up to 13%, with PPV ranging from 0.529 to 0.100.^26-28,30,32-34^ By contrast, the CSF flow velocity study showed the highest conversion rate (16%) and the lowest PPV.^31^ Fundamentally, the conversion rates lie within the same range as the reoperation rates previously reported in dogmatic PFD-only studies, which range from 0% to 42%,^18,42-44^ calling into question the real benefit of iUS.

### Spatial Dynamics of the Craniocervical Junction

Another challenge in patients with CM-1 is the dynamic at the craniocervical junction, which is significantly influenced by head, neck,^45^ and body positioning.^46,47^ During daily life, the head is predominantly upright, whereas conventional diagnostic imaging is conducted supine, and intraoperative measurements are obtained in a prone, head inclined, and rigid position. This mismatch raises concerns that measurements taken in the operating room may not reflect the real-life spatial conditions at the craniocervical junction and could either lead to an overestimation or underestimation of decompression. This limitation has been stressed previously in relation with the poor predictive value of intraoperative magnetic resonance imaging for determining whether to open the dura in CM-1 surgeries.^47^

### Future Directions: Standardizing iUS and Guiding Clinical Practice

**FIGURE 3.**
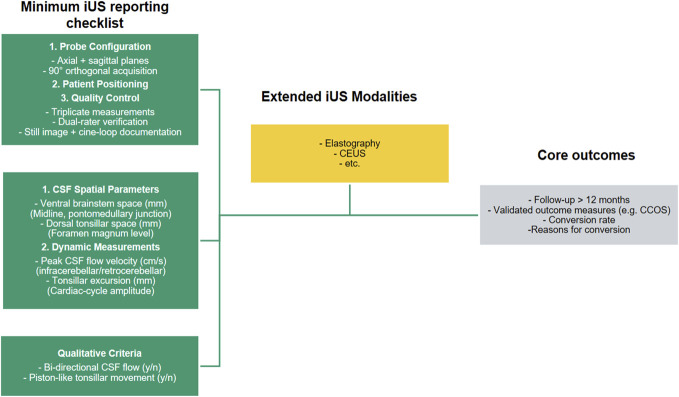
Proposed reporting framework for iUS studies, highlighting a minimum reporting checklist (green), possible emerging techniques (yellow), and relevant outcome measures (grey). Specific details include CSF space measurements ventral to the brainstem and dorsal to the tonsils, dynamic parameters such as peak CSF flow velocity and tonsillar excursion amplitude and operational definitions for qualitative criteria supported by cine-loop examples. Emerging modalities could be elastography for tissue stiffness and perfusion imaging for microvascular assessment (both have not yet been reported in Chiari surgery but are proposed based on applications elsewhere in neurosurgery^48,49^). CCOS, Chicago Chiari Outcome Scale; CEUS, contrast-enhanced ultrasound; CSF, cerebrospinal fluid; iUS, intraoperative ultrasound; n, no; y, yes.

To address the absence of standardized acquisition, measurement, and reporting protocols, we propose a reporting framework (Figure 3) that emphasizes quantitative assessment while incorporating clear definitions for qualitative features.

Core requirements include documentation of acquisition parameters, CSF space measurements at defined locations, and dynamic metrics such as CSF flow velocity and tonsillar excursion amplitude. Measurements should be obtained in triplicate, averaged, and confirmed by 2 raters. Qualitative observations, including bidirectional flow or piston-like motion, must be operationally defined and supported by representative imaging.

Validation requires ≥12-month FU,^22^ validated outcome measures such as the Chicago Chiari Outcome Scale^50^ and detailed documentation of conversion surgeries. This framework consolidates all criteria currently used in iUS, while recognizing that none are sufficient alone. By mandating comprehensive reporting, it provides the foundation for future pooled analyses and identification of the most relevant parameters.

### Limitations

Our literature review has several limitations. The included studies are heterogeneous in demographics, outcome measures, and FU periods, affecting consistency and generalizability. Owing to this heterogeneity, reliable subgroup comparisons could not be performed. In addition, the variable and short FU durations reflect real-world reporting but limit the evaluation of long-term prognostics. Most lacked prospective designs, limiting definitive conclusions. In addition, reliance on published data for iUS diagnostic value means findings should be viewed as estimates rather than precise values. We did not analyze surgical details such as dural graft use, dura scoring, or splitting. As some patients may avoid further surgery despite persistent symptoms, conversion rates and PPV likely overestimate iUS benefit, limiting them as outcome measures. Furthermore, in patients with symptom relief after PFD, recurrence does not always indicate failure, as new bone formation or scarring can occur, factors we could not reliably assess. As this is not a meta-analysis, synthesized data must be interpreted cautiously.

## CONCLUSION

The available evidence does not support iUS as a reliable method for intraoperative decision making regarding bone-only PFD for CM-1, primarily due to the heterogeneity of its application and the absence of standardized assessment criteria. Despite its frequent use, its accuracy in determining the sufficiency of bone-only PFD remains uncertain, making it unreliable for definitive surgical decisions. To address this gap, we propose a reporting framework that consolidates all currently reported criteria, recognizing that none are yet sufficient in isolation. Adoption of such an approach could enable to identify the most relevant parameters more efficiently. Further research with homogeneous comparative cohorts is needed to enhance reproducibility, determine whether iUS reduces conversion surgery, and clarify its impact on clinical outcomes.

## COMMENTS

This article offers a systematic review of intraoperative ultrasound (iUS) in the surgical management of Chiari 1 malformation, evaluating its clinical utility and highlighting a gap in the literature. Despite the increasing use of iUS to inform whether bone-only posterior fossa decompression (PFD) suffices or if duraplasty (PFD+) is warranted, the authors reveal that current applications of iUS are undermined by inconsistent criteria and reporting standards. Their analysis—spanning 9 studies and 844 patients—shows substantial heterogeneity in iUS protocols, outcome definitions, and thresholds for surgical conversion, which fundamentally limits the test's reliability and the interpretability of published results.

The review emphasizes that sensitivity and specificity for iUS remain incalculable due to methodological limitations, challenging the widespread perception that iUS can accurately guide intraoperative decision-making. The authors propose a comprehensive reporting framework for future iUS studies, advocating for standardized acquisition protocols, quantitative and qualitative assessment measures, and robust outcome reporting, including at least 12-month follow-up using validated scales. By consolidating current criteria and highlighting emerging technologies such as elastography and perfusion imaging, the framework seeks to lay the groundwork for meaningful data pooling and consensus-building.

The review both exposes the limitations of iUS as currently practiced and suggests path forward, calling for objective iMRI criteria and reliable outcome measures to clarify iUS's role in tailoring Chiari 1 decompression.

**David Houston Harter**

*New York, New York, USA*
